# Quantitative Analysis of Cell and Tissue Shape During Mouse Cranial Neural Tube Closure

**DOI:** 10.21769/BioProtoc.5711

**Published:** 2026-06-05

**Authors:** Kristina A. Borys, Eric R. Brooks

**Affiliations:** Department of Molecular Biomedical Sciences, College of Veterinary Medicine, North Carolina State University, Raleigh, NC, USA

**Keywords:** Neural tube closure, Cranial tissues, Mouse development, Quantitative image analysis, Segmentation

## Abstract

Neural tube closure is a critical process that transforms the neural plate, an open epithelial tissue, into the closed tube that serves as the structural basis of the central nervous system. Defects in this process are among the most common and severe developmental diseases in the human population, with failures in cranial closure accounting for approximately one-third of total defects. However, the cell and tissue mechanisms that drive cranial closure remain opaque relative to the better studied process of spinal closure, in large part due to the unique challenges in characterizing cranial tissues. Here, we present protocols for quantifying cell dynamics and tissue-level remodeling events that enable highly spatiotemporally resolved investigations of the causes of cranial closure defects in mouse embryos. These include brightfield morphometric approaches, fluorescent staining and confocal imaging, and quantitative pipelines to analyze these image-based datasets. At the conclusion of these approaches, users will be able to quantify several parameters of overall tissue shape in the cranial neural tissues and produce rich quantitative datasets about cell-level parameters, particularly apical cell area. These can be used to identify correlative and causative differences between mutants and control embryos. Given their flexibility, many of these approaches can be generalized to other tissue morphogenetic contexts.

Key features

• Provides flexible and quantitative pipelines for cell and tissue-scale morphometrics, which can be expanded to many morphogenetic problems.

• Presents robust mounting and imaging methods for cranial tissues.

• Allows assessing the role of cellular-scale remodeling events in deforming tissues during cranial closure and how these are changed in mutants.

## Graphical overview


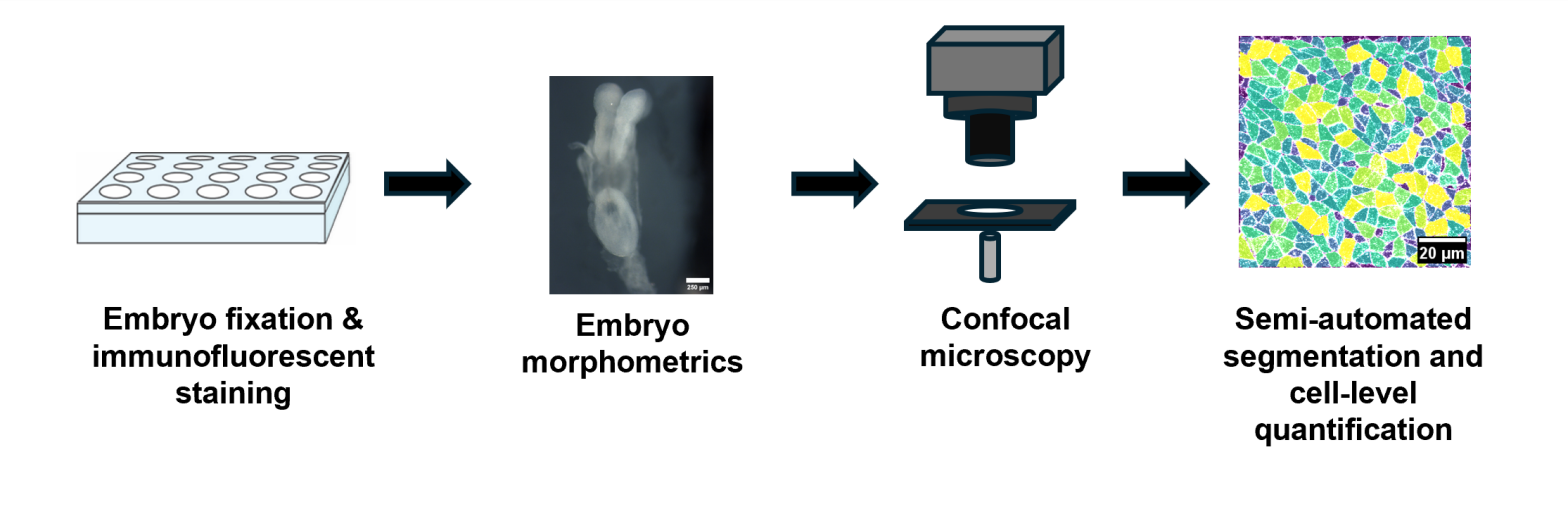


## Background

Neural tube closure converts the early neuroepithelial tissues—a sheet of cells also known as the neural plate—into the closed tube that provides the structural basis of the brain and spinal cord. Defects in this process are among the most common and deleterious developmental defects in humans and occur in specific regions along the head-to-tail axis [1–4]; defects specifically in cranial closure account for approximately one-third of reported human neural tube defects [5,6]. Several cellular mechanisms have been identified as drivers of closure in spinal tissues, including apical constriction and convergent extension (reviewed in [7–11]). However, less is known about the mechanisms of cranial closure, due in part to the challenges presented by the larger size and highly curved nature of the anterior neuraxis. Recently, we and others have developed approaches to adapt to these challenges, allowing the examination of cranial closure at high spatiotemporal resolutions, leading to increased understanding of the mechanisms of closure in more posterior domains [12–20]. Given the large library of mouse mutants and the vast array of environmental toxicants that can disrupt cranial closure [21–25], these approaches will be central to understanding the cellular and tissue-level drivers of these defects.

Here, we present our workflows for quantitative analysis of cell and tissue-level properties of the cranial neural plate during closure. We have used these approaches to show the role of Sonic hedgehog and Wnt signaling in patterning apical constriction, as well as the role of Wnt signaling in setting the scale of cranial neural tissues [12-14]; similar approaches from other groups have also been used to investigate the mechanics of apical constriction, apical-basal polarization, and pseudo-stratification within cranial tissues [15,17,18,20]. While we include specific approaches to enable analysis of the large and highly curved cranial tissues, large portions of the downstream workflows we present here can also be readily adapted to the analysis of other problems in tissue morphogenesis in the mouse embryo or other species contexts. Thus, these pipelines represent generalizable tools for investigating the molecular, cellular, and tissue-level drivers that reshape tissues for function during embryonic development.

## Materials and reagents

**Biological materials**

1. Mouse embryos at timepoints between embryonic day 7.5 (E7.5) and E8.75 (0–9 somites) with resected yolk and amniotic sacs fixed in 4% paraformaldehyde (see section A for recommended fixation conditions and embryo staging)

**Reagents**

1. 10× phosphate-buffered saline (PBS) (Fisher, catalog number: BP399-500); an equivalent solution can be prepared from salts, as in [26]

2. Triton X-100 (ThermoFisher, catalog number: A16046.AP)

3. 4% paraformaldehyde in PBS (e.g., Fisher, catalog number: AAJ61899AP), aliquoted in 1 mL tubes and stored at -20 °C until use or up to 1 year

4. Bovine serum albumin (BSA) (e.g., Fisher, catalog number: BP9703100)

5. Agarose, low melt (ThermoFisher, catalog number: H26417.14)

6. Rabbit anti-N-cadherin (Cell Signaling Technology, catalog number: 13116S)

7. Fluorescent conjugated anti-mouse secondary antibodies (e.g., Donkey anti-mouse Alexa488) (Invitrogen, catalog number: A32787)

8. Fluorescent conjugated phalloidin (e.g., Alexa546 conjugated phalloidin) (ThermoFisher, catalog number: A22283)

**Solutions**

1. 1× PBS + 0.1% Triton X-100 (PBTriton) (see Recipes)

2. Blocking solution (see Recipes)

**Recipes**

**1. PBTriton**

**Table:** 

Reagent	Final concentration	Quantity or volume
10× PBS	1×	100 mL
Triton X-100	0.1% (v/v)	1 mL
Deionized or reverse osmosis water	n/a	899 mL
Total		1,000 mL

This solution is stable for up to one year at 4 °C.

**2. Blocking solution**

**Table:** 

Reagent	Final concentration	Quantity or volume
10× PBS	1×	100 mL
Triton X-100	0.1% (v/v)	1 mL
BSA	10% (w/v)	100 g
Deionized or reverse osmosis water	n/a	899 mL
Total		1,000 mL

As an alternative, you can buy premade 10% BSA in PBS blocking solution (e.g., ThermoFisher, catalog number: 37525) and add 0.1% Triton X-100. This is stable and—with the addition of detergent—exhibits no biological contamination in our experience for up to one year at 4 °C. If biological contamination is a concern, a small amount of preservative (e.g., 0.05% sodium azide) can be added without impacting downstream use.

Make solutions in 1,000 mL glass bottles. Triton should be added after diluting the 10× PBS stock with water. At stock concentration, it is quite viscous, so cutting a small portion off a pipette tip can help in transferring. Use a magnetic stir bar and plate to thoroughly incorporate Triton into the solution (~30 min).

**Laboratory supplies**

1. 35 mm untreated plastic or glass Petri dishes (e.g., Fisher, catalog number: FB0875711YZ, though this can be any small Petri dish)

2. Plastic transfer pipettes (e.g., Fisher, catalog number: 13-711-7M)

*Note: Ensure that the mouth of the pipette is large enough in diameter to allow the embryo to enter the pipette; if the diameter is too narrow, a pair of scissors can be used to cut off the tip.*

3. 24-well untreated plastic well plates (e.g., Genesee Scientific, catalog number: 25-103) or 2 mL Epi tubes (e.g., Genesee Scientific, catalog number: 24-283) for fixing and staining embryos

4. 25 mm circular #1 or #1.5 cover glasses for imaging dishes (e.g., Fisher, catalog number: 50-948-978)

5. Square or rectangular cover glass cut or broken into <10 mm squares

6. Molykote vacuum grease (Fisher, catalog number: 14-635-5D)

7. 1 mL Luer Lock syringe (e.g., Fisher, catalog number: 14-823-30) with a 200 μL pipette tip screwed onto the Luer Lock

## Equipment

1. Watchmakers forceps, ideally #55 or #5 (e.g., Fine Science Tools, catalog number: 11295-51)

2. Orbital shaker or nutator

3. Attofluor reusable aluminum imaging dishes (ThermoFisher, catalog number: A7816)

4. Dissecting stereomicroscope or macroscope equipped with brightfield camera (e.g., Zeiss Lumar, model: V12)

5. Confocal laser scanning microscope in inverted light path configuration and equipped with lasers appropriate to the fluorescent conjugates of your antibodies (e.g., Zeiss, model: LSM 980)

6. Computer for image analysis

## Software and datasets

1. FIJI/ImageJ (https://fiji.sc/; NIH, and worldwide contributors, see [27,28], free to use)

2. The MorphoLibJ plugin for FIJI/ImageJ (https://github.com/ijpb/MorphoLibJ, INRA-IJBP modeling lab, see [29], free to use)

3. Cellpose (https://www.cellpose.org/, see [30], free to use); alternatively, cell-wise segmentation program (e.g., SeedWater segmenter, David Mashburn/Hutson Lab, see [31], free to use)

*Note: We routinely update our FIJI/ImageJ distributions, the MorphoLibJ plugin, and the Cellpose software; to date, it has not impacted the workflow presented below. As of this writing, the versions we used were: FIJI/ImageJ- ImageJ 2 16.0/1.54p (running on Java 1.8.0_172); MorphoLibJ-1.6.5; Cellpose-4.1. For Cellpose, we recommend starting with the pretrained CP (cyto fluorescent) model and retraining it on manually corrected data from 10 embryos for any given stain to increase the accuracy of the automatic segmentation.*

## Procedure

*Notes:*

**Figure 1.**
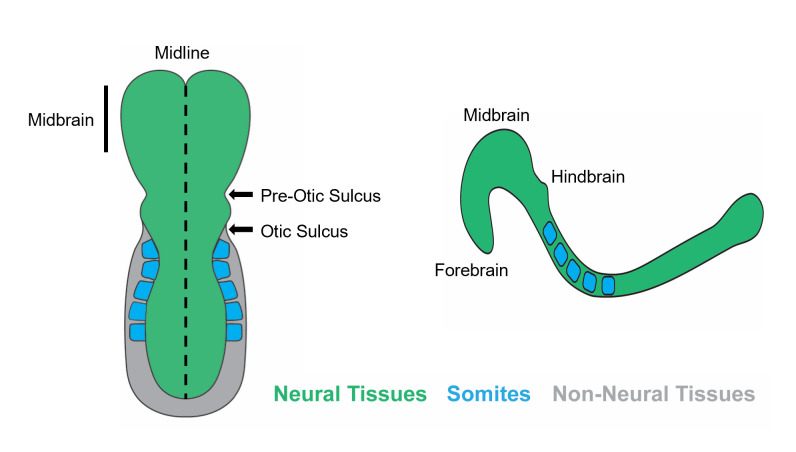
Schematic representation of an E8.5 mouse embryo with 5 somites (blue color) in dorsal (left) and side-on (right) views. Key morphological landmarks, including the pre-otic and otic sulci, are shown.

*1. Embryo staging: In the following procedures, we use somite number to stage embryos. Somites are visible blocks of mesodermal tissue adjacent to the neural tissues, beginning at the level of the posterior hindbrain (see Figure 1). These tissue blocks begin appearing between E7.5 and E8.0, with a new somite addition every ~2 h. For more detailed staging information, including additional morphological criteria corresponding to these stages, see [26,32].*

*2. Embryo dissection: Prior to fixation, embryos are dissected in ice-cold PBS as outlined in [26], with both the yolk sac and the amnion being resected. For embryo genotyping, we generally use the allantois and/or a small piece of the posterior embryo. This tissue can be removed concurrently with brightfield imaging so as not to interfere with morphological characterization.*

**A. Fixation and immunostaining of mouse embryos at neural plate stages**

**Figure 2.**
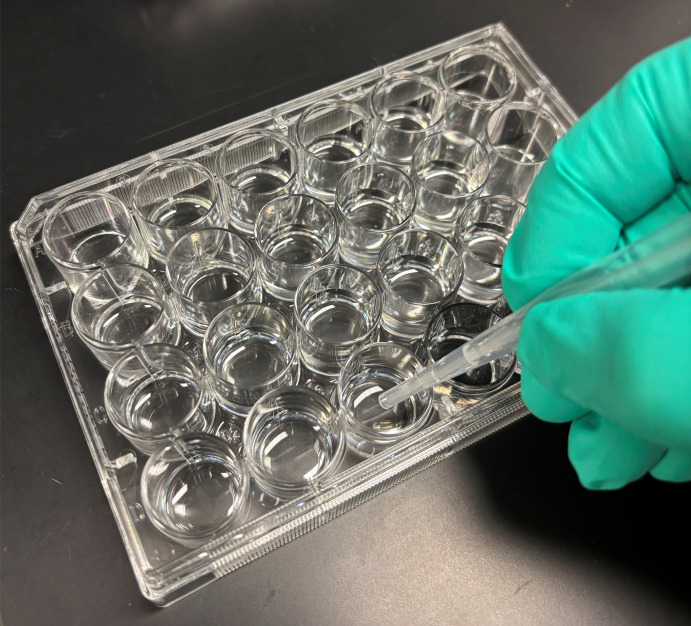
Transferring embryos from a 24-well plate using the transfer pipette technique

*Note: We prefer to do our staining in 24-well plates (see Figure 2). In this method, fixation in 1 mL of 4% paraformaldehyde in PBS occurs in the first well; then, embryos are transferred to a new well at each wash and stain step. We also prefer to stain all embryos together and then separate them and take small pieces of the allantois or posterior tissues for genotyping. This helps control against the significant embryo-to-embryo variability that can occur if each embryo is stained separately. All steps below could alternatively be performed in 2 mL Epi tubes, either treating all embryos as a group or separating them after genotyping. Up to 12 (and possibly more) embryos can be stained together without loss of signal, in either a well or an Epi tube. All wash and stain steps should be performed either on an orbital shaker (set to ~60 rotations per minute) or a nutator.*

1. After dissection (see [26] for dissection protocols), fix embryos in 4% paraformaldehyde for either 1 h at room temperature or overnight at 4 °C; 500 μL of fixative in the well of a 24-well plate is sufficient. In our experience, the overnight fixation at a lower temperature provides a better signal-to-noise ratio upon staining with antibodies. **(Pause point)**

2. When transferring embryos between wells, the safest method is to use your thumb and finger to squeeze the pipette near the mouth and only pull in enough liquid to hold the embryo (Figure 2).

**Caution:** Once the embryo is in the pipette, maintain constant pressure until you have placed the mouth below the surface of the liquid in the new well. If the embryos do not freely enter the liquid, they can be dislodged by gently shaking or stirring the submerged pipette mouth.

3. After fixation, wash the embryos three times in PBTriton, for 30 min per wash, at room temperature (use 1 mL of PBTriton in one well of a 24-well plate for each wash).

4. Place embryos in blocking solution (500 μL in a well of a 24-well plate is sufficient) and incubate at room temperature for 1 h.

5. Make up 500 μL of blocking solution containing primary antibodies at the desired concentrations. For anti-N-cadherin, we use a 1:500 dilution. Incubate embryos in antibody solution for either 1–2 h at room temperature or overnight at 4 °C. In our experience, overnight incubation frequently gives better signal-to-noise ratios, but this depends on the antibody. **(Pause point)**

6. Wash the embryos three times in PBTriton for 30 min per wash at room temperature.

7. Make up 500 μL of blocking solution containing secondary antibodies and counterstain at the desired concentrations. We use donkey anti-rabbit AlexaFluor488 conjugated secondaries at 1:500 and a 1:1,000 dilution of AlexaFluor546 conjugated phalloidin. Incubate the embryos in this solution for either 1–2 h at room temperature or overnight at 4 °C. **(Pause point)**

**Caution:** From this step onward, embryos should be protected from light by covering the plate or tube with aluminum foil to minimize photobleaching.

8. Wash the embryos three times in PBTriton for 30 min per wash at room temperature.

9. Embryos can be stored in PBTriton at 4 °C, with the well plate or Epi tubes covered with aluminum foil, for up to two weeks. Imaging earlier in this window is preferred, but we notice only a slight signal degradation onset at 2 weeks. After 2 weeks, tissue morphology begins to slowly degrade.

**B. Brightfield embryo imaging**

**Figure 3.**
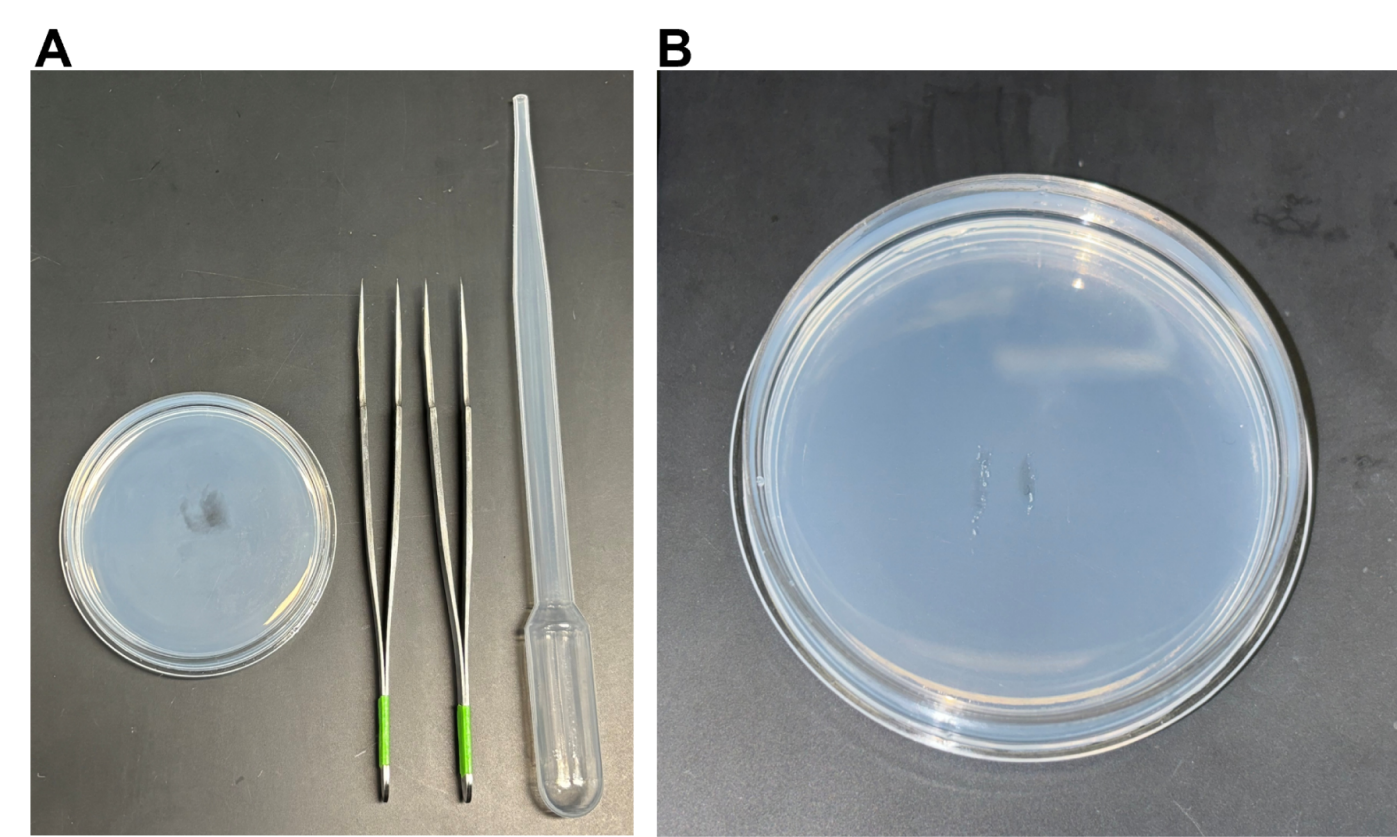
Brightfield imaging tools. (A) From left to right: 2% agarose in a 35 mm plastic Petri dish filled with PBTriton, two #55 watchmaker's forceps, and a plastic transfer pipette. (B) Close-up of divots/gouges in agarose pad.

**Figure 4.**
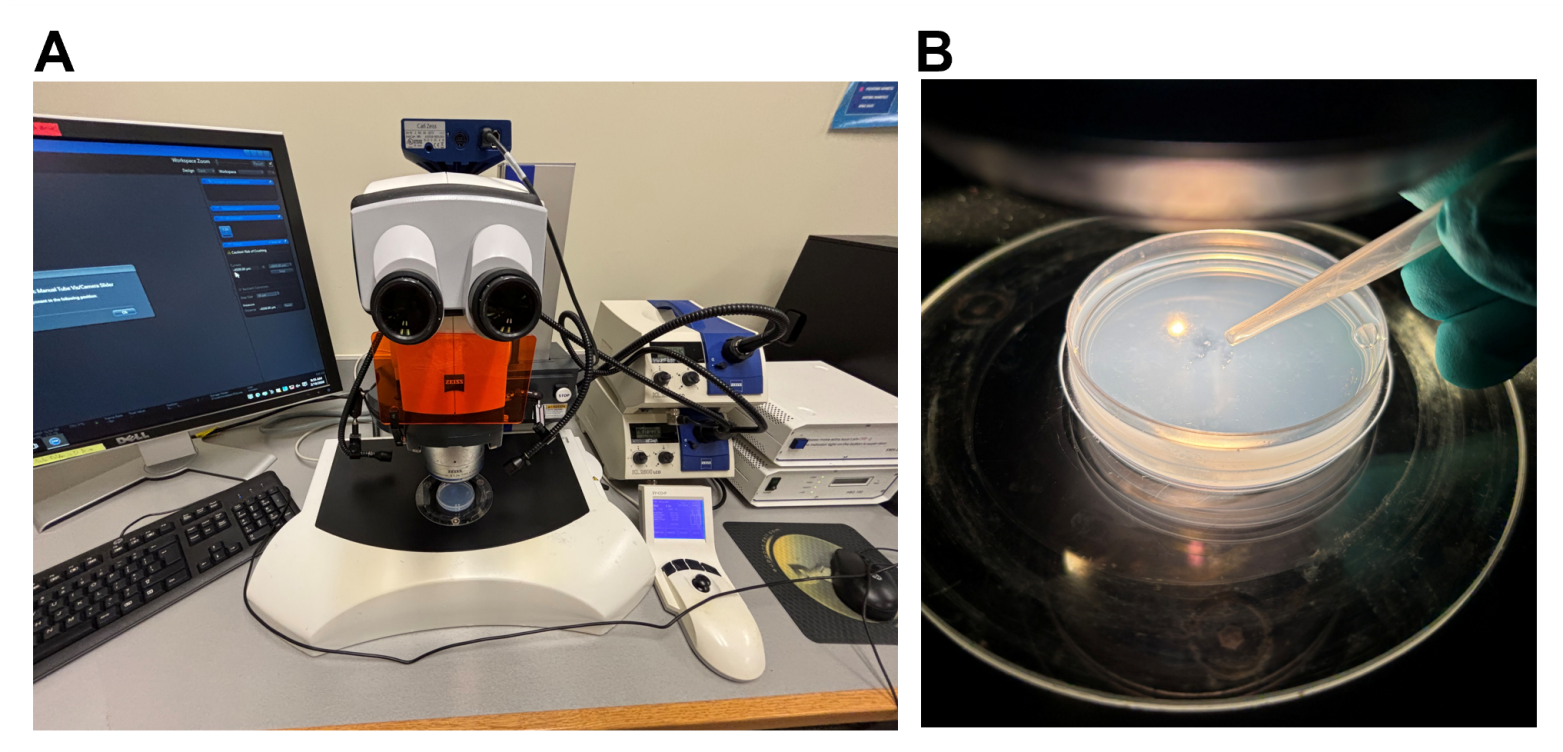
Brightfield imaging setup. (A) Agarose Petri dish is placed under Zeiss Lumar V12. (B) Close-up of agarose Petri dish containing an embryo in the left divot.

1. Prepare a brightfield imaging dish by pouring molten 2% agarose into a 35 mm plastic or glass Petri dish until it is approximately halfway full. Then allow the agarose to cool and solidify (Figure 3). Dishes can be reused for several months if kept damp and refrigerated. As an alternative, colored modeling clay could be used as a base for the imaging dish. This increases background contrast but also has a higher chance of leaving unwanted residue on the embryos.

2. Using a brightfield microscope (Figure 4), gather the fixed embryos imaging dish, forceps, one or two transfer pipettes, and a small volume of PBTriton (a 50 mL Falcon tube suffices).

**Caution:** Ensure the microscope, camera, and light sources—ideally freely deformable gooseneck lamps—are on and warmed up.

3. Pour enough PBTriton into the imaging dish to provide a sufficient liquid layer to comfortably cover embryos. Place the imaging dish in the center of the field of view and, using a transfer pipette, carefully transfer one embryo into the dish.

**Caution:** The safest method is to use your thumb and finger to squeeze the pipette near the mouth and only pull in enough liquid to hold the embryo (Figures 2 and 4B). Once the embryo is in the pipette, maintain constant pressure until you have placed the mouth below the surface of the liquid in the dish.

**Figure 5.**
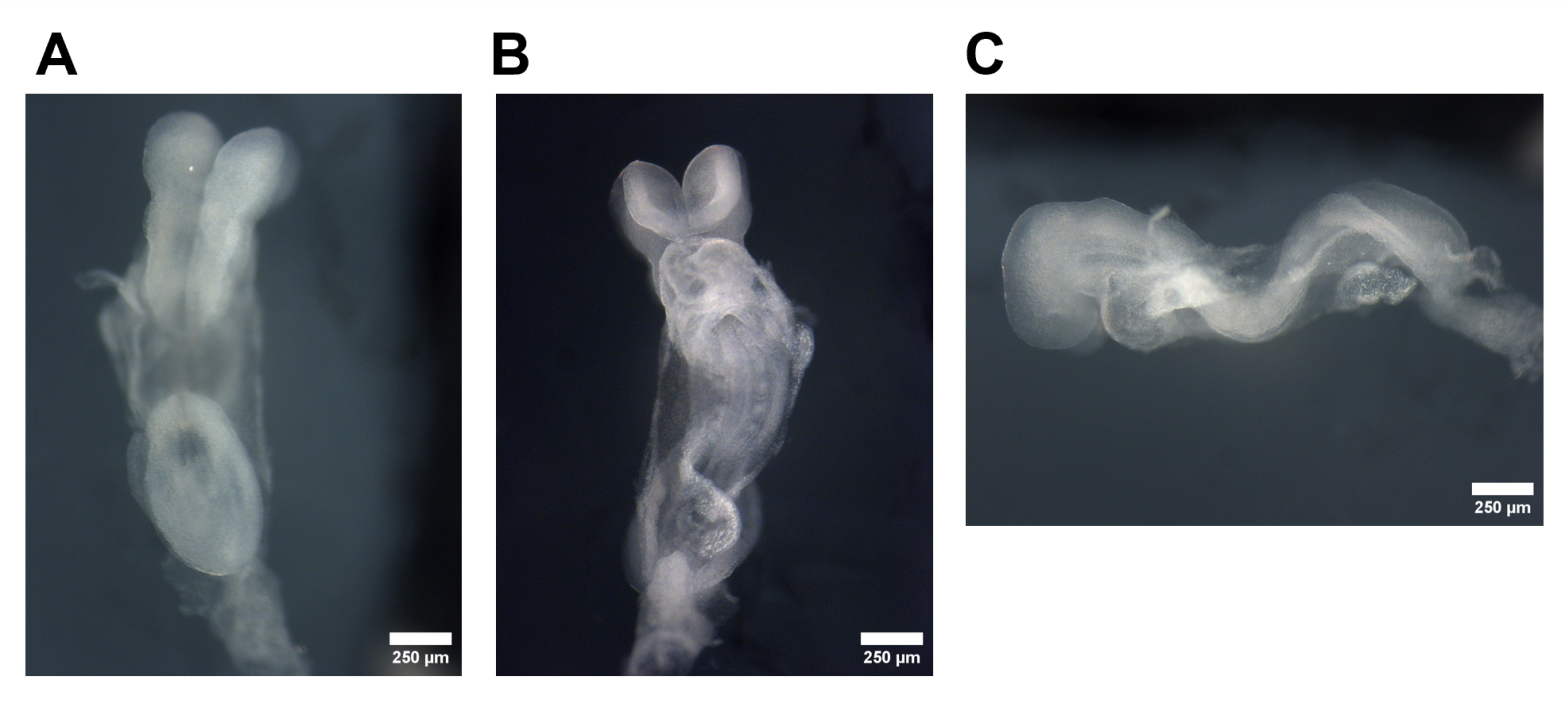
Brightfield orientations of embryos. Embryo in dorsal (A), ventral (B), and side (C) orientations. In this protocol, we will use the dorsal and side orientations for quantification. The ventral orientation is useful for analyzing forebrain morphology and ensuring data reusability for future analyses.

4. Using your forceps to move and roll the embryo, take images in three positions; first, lay the embryo gently on its ventral surface so that the camera is looking straight down at the dorsal surface of the neural folds (Figure 5A). Second, turn the embryo over so that it is resting on its dorsal surface to capture the ventral orientation (Figure 5B). While this ventral view is not used in any of the quantifications below, it is useful for examining forebrain morphology and ensures you have a full set of views of the embryo, should you need to return to it later for further analysis. Finally, turn the embryo onto either its left or right side (Figure 5C). In our work, we have tried either consistently choosing one side for each embryo in a litter, or alternating sides based on what allows the embryo to rest more readily in an orthogonal view. We have not found any inter- or cross-litter differences between these two methods.

**Caution:** Note that the embryos are fairly buoyant and will move with any disturbance to the liquid or dish. To hold embryos in position while you take pictures, you can create small gouges/divots in the agarose surface with your forceps and rest the embryo in these (Figure 3B). These gouges should be roughly the same size (or smaller) than the embryos (~0.5–1.5 mm).

5. Make sure to take images at a reasonable magnification, i.e., one where you can distinguish the anatomical structures of the embryo while still seeing the totality of the tissue. For example, we routinely use ~40× total magnification, which, with a frame size of 1,388 × 1,040 pixels, translates to a pixel size of ~1.7 μm^2^. Ensure that magnification stays constant over all images and embryos.

**Critical:** If the microscope you are using does not automatically calculate the real scale of the field of view (and include it in the image file), make sure to take a photograph of the millimeter hash marks of a ruler or some other object that can be used to set the scale of your images during analysis.

6. Once all embryos have been imaged in this manner, the agarose dish can be stored in a refrigerator for later use or discarded.

**C. Mounting and whole-mount confocal microscopy of the cranial neural folds**

*Note: This protocol describes mounting in reusable aluminum Attofluor imaging dishes (Figure 5D). However, similar steps can be used with disposable glass-bottom 25 mm dishes. Alternatively, a temporary imaging chamber can be assembled from a Petri dish and a coverslip, as described in [33].*

**Figure 6.**
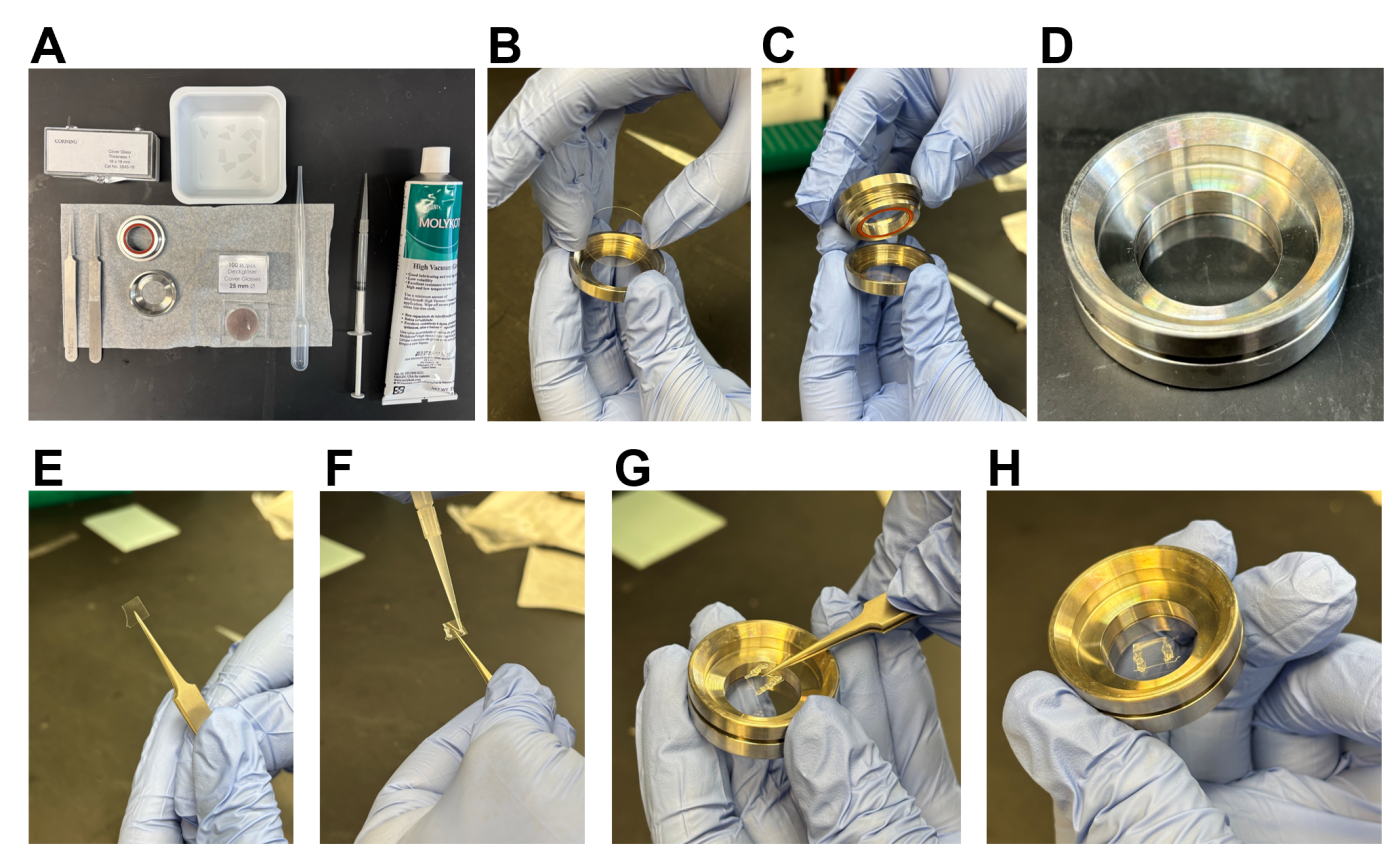
Preparing a whole-mount imaging dish. (A) Materials used (from top to bottom, left to right): 18 × 18 mm cover glass, cover glass pieces, two #55 watchmaker forceps, aluminum Attofluor imaging chamber top and bottom, 25 mm round coverslips, transfer pipette, 1 mL syringe filled with Molykote vacuum grease, and Molykote vacuum grease. (B) A round coverslip is placed at the bottom of the imaging dish. (C–D) The top of the imaging dish is screwed into the bottom piece finger-tight. (E) The tips of forceps are used to hold a small piece of cover glass, and (F) a thin line of grease is applied to two edges. (G) The greased side of the cover glass is placed at an angle onto the cover glass and (H) secured.

1. Place a 25 mm round coverslip in the depression in the bottom piece of the Attofluor chamber and screw the top piece on until finger-tight (Figure 6B–D). Dust both sides of the coverslip with a Kimwipe or piece of lens paper.

2. Using a 1 mL syringe filled with Molykote vacuum grease and capped with a 200 μL pipette tip, place small dabs or lines of grease on the two parallel edges of a small piece of cover glass (Figure 6E–F).

3. Place the greased cover glass on top of the coverslip in the Attofluor at an angle so that the embryo can be placed under it (Figure 6G–H). Gently push down on the edges of the cover glass to create a seal with the underlying coverslip.

4. Add 1 mL of PBTriton to the Attofluor chamber.

**Caution:** Check for leaks by placing the chamber on top of a Kimwipe or paper towel. If the chamber contains large dust granules/fibers, you can gently remove and replace the PBTriton.

**Figure 7.**
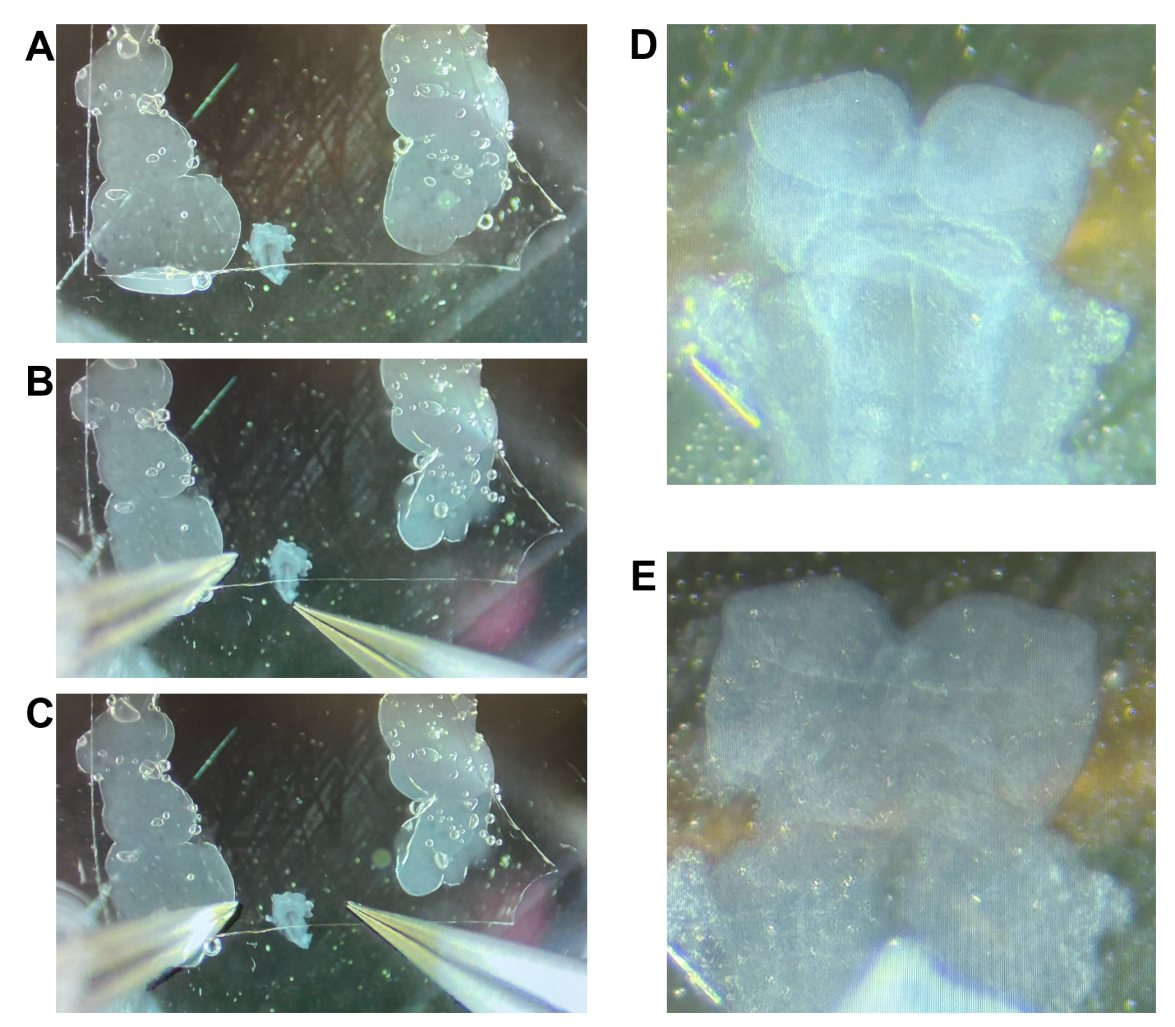
Mounting an embryo for whole-mount imaging. (A) The embryo is placed under the cover glass shard and then (B) positioned using the tips of the forceps. (C) Forceps apply pressure to both sides of the embryo to secure it in place. (D) Image of a normally mounted embryo and (E) an image of an over-compressed embryo.

5. Place the embryo with the dorsal surface of the cranial neural folds down against the coverslip and slide it under the piece of cover glass (Figure 7A, B). Using forceps on either side, gently push the piece of cover glass down until the embryo is lightly sandwiched between the two pieces of glass (Figure 7C).

**Caution:** Do not over-compress the embryo, as that can lead to tearing of the midline or other confounding tissue deformations. Over compression is apparent when the embryo appears to be stretched (Figure 7D, E), bulging, or flattened (like a pancake). Overly compressed embryos will not return to their original shape when the downward pressure of the cover glass piece is relieved, whereas appropriately mounted embryos will.

**Figure 8.**
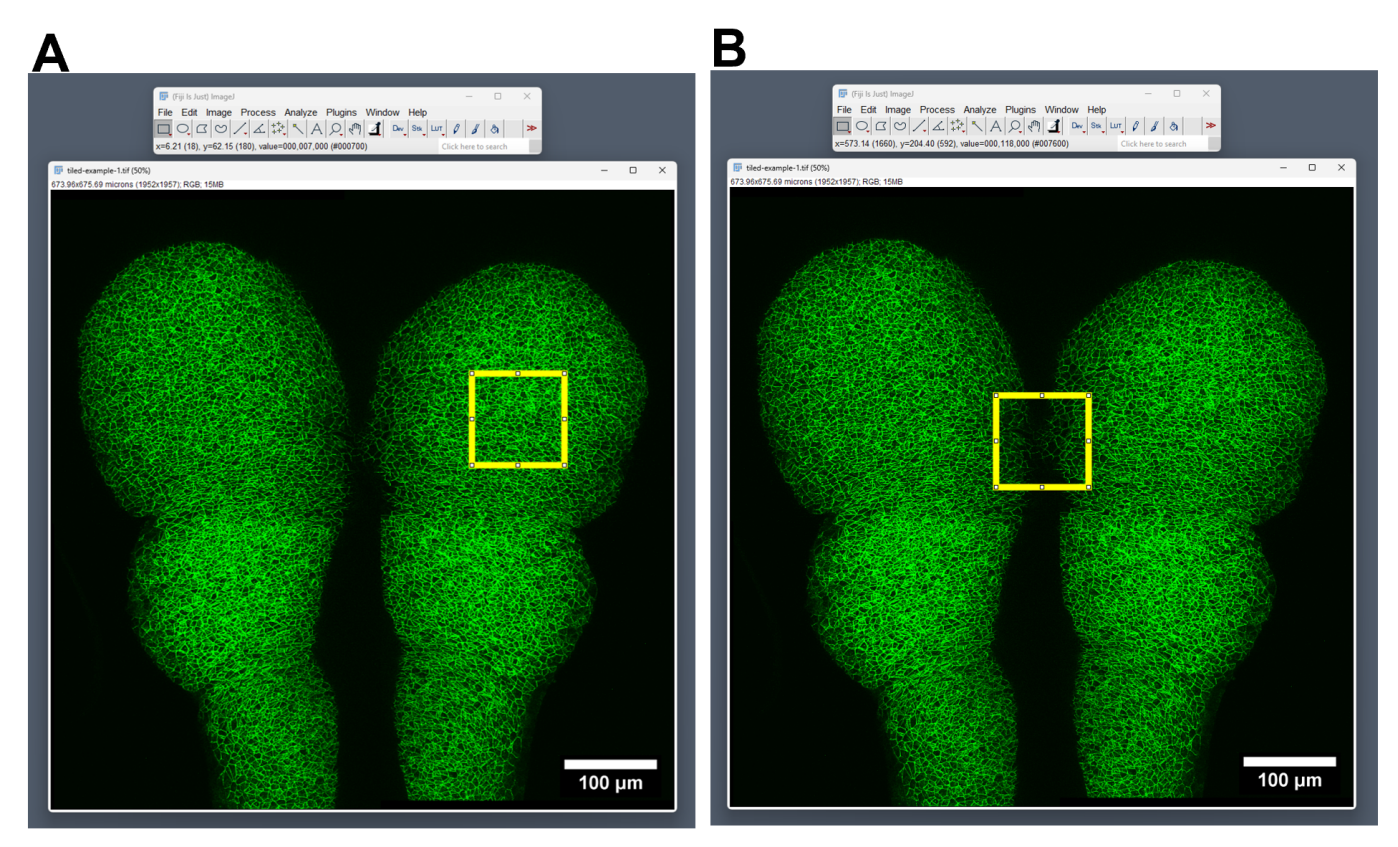
Tiled cranial neural folds stained for N-cadherin. Embryos (~7 somites) with 100 µm × 100 µm regions of the right lateral midbrain (A) and midline (B). These represent the regions of interest (ROIs) we would use for analysis.

6. Place the dish with the mounted embryo on the confocal microscope and set up the acquisition parameters to match the fluorophores used in your staining. For analyzing individual cell morphology, we acquire images using a 40× oil immersion objective. We generally use a frame size of 1,024 × 1,024 pixels, with a pixel scale of 0.35 μm^2^. To keep image properties comparable across a litter, we briefly look at 2–3 embryos to assess how uniformly they stained and set acquisition parameters such as laser power and detector gain to allow acquisition without oversaturation of more than a handful of pixels in each channel across those embryos. We then keep these parameters constant throughout the litter. When acquiring, we use a bi-directional scan and frame acquisition approach to collect all channels at a single z-plane before moving to the next z-position.

7. Acquire a z-stack that encompasses the whole depth of the cranial neural plate centered within the lateral domain of the future midbrain (the region shown in Figure 8A). If desired, you can also use a tiling or multiposition plugin to acquire the overall tissue morphology (similar to the overall image shown in Figure 8). On our Zeiss LSM 980, we use 10% overlap when acquiring tiled images, but consult your user manual for your system’s recommended overlap. Alternatively, if you do not have tiling capability on your confocal, you can simply acquire an additional z-stack centered on the midline (the region highlighted in Figure 8B).

8. Make a maximum intensity projection of the entire volume of the z-stack while ensuring to save both the original z-stack and the projection for reuse. For each embryo, we analyze either one 100 × 100 μm^2^ region of interest (ROI) from the left lateral neural fold (Figure 8A) or paired ROIs from both the left and right lateral neural folds. However, we have not noticed significant differences in our final analysis when analyzing only single vs. paired lateral regions; therefore, we recommend analyzing a single region. In either case, only a single 100 × 100 μm^2^ ROI of the midline is analyzed.

9. Continue imaging until you have made it through all the embryos you intend to analyze. You can reuse the mounting dish for 2–3 embryos, but the grease will eventually lose adhesion; at that point, a new dish should be made. To reuse a dish, use forceps to gently lift the edge of the cover glass piece upward, retrieve the imaged embryo, and place a new one under the glass. Repeat mounting as above.

## Data analysis

**A. Tissue morphometrics from brightfield images**

*Note: Here, we will use NIH ImageJ (or the FIJI redistribution of the same software, which also comes preinstalled with many helpful plugins) to derive quantitative values for various properties of the tissue. FIJI/ImageJ can be readily installed on Windows, Macintosh, and Linux operating systems (see Software). For general help with FIJI/ImageJ, please see the user guide and wiki (* *https://imagej.net/* ).

**Figure 9.**
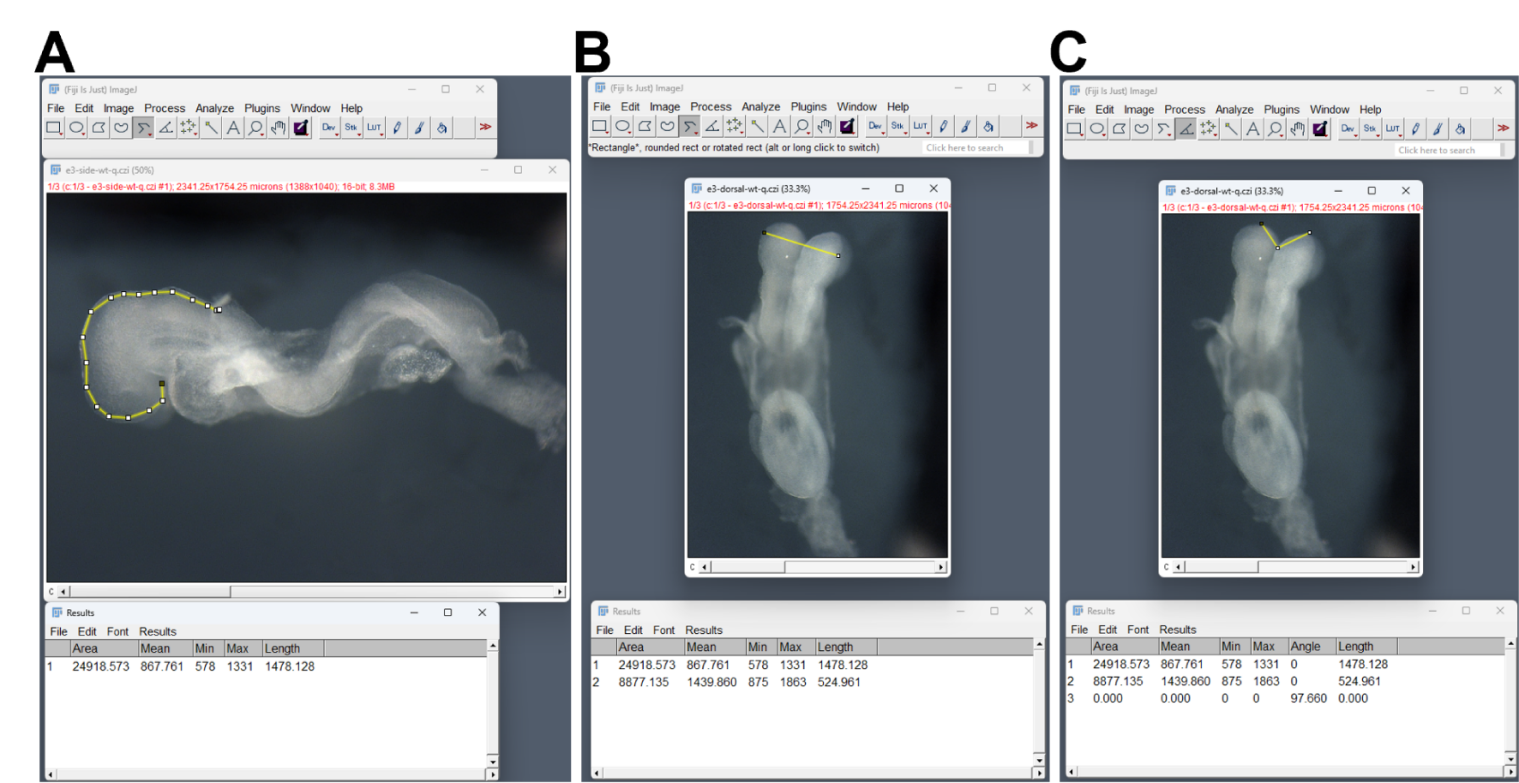
Tissue morphometrics using brightfield images. (A) Side view of an embryo with a segmented yellow line measuring the length. (B) Dorsal view of an embryo with a straight yellow line measuring the width. (C) Dorsal view of an embryo with the yellow angle measuring the angle of elevation.

1. Open Fiji/ImageJ.

2. Import a brightfield image (you can either use the *File* menu or drag the file directly onto the FIJI/ImageJ task bar).

3. If your image does not have metadata about the scale of the image in real terms, you will need to set this manually. If so, open your image of a ruler or other object with known dimensions and measure how pixels correspond to a known length, e.g., the distance in pixels between two adjacent millimeter hashes on a ruler can be found by using the *line tool* to draw a perpendicular line between them and then running the *Measure* function under the *Analyze* menu. Divide the known length by the number of pixels to get the pixel scale value and enter that value in the *Set Scale* window (accessed under the *Analyze* menu).

4. To measure the width of the cranial neural tissues, you can use the *line tool* to draw a straight line perpendicular to the head-to-tail axis at the widest point of the future midbrain in your dorsal image (Figure 9B). Then, use the *Measure* function in the *Analyze* menu. Note that, by default, FIJI/ImageJ keeps a running tally of your measurements (the *Results* window is shown at the bottom of all images in Figure 9), so you analyze multiple embryos and then copy the values to a spreadsheet at the end. Note that this approach can be used to measure width at any point along the neuraxis if you wish to compare, e.g., midbrain and hindbrain width.

5. To measure the length of the cranial neural tissues, open your side-on view of the embryo and use the *segmented line tool* (right-click on the *line tool* and select the *segmented line*) to draw a connected series of line segments along the cranial neuraxis, from the level of the otic sulcus to the furthest anterior extent of the cranial neural tissues (Figure 9A); then, use the *Measure* function. It is important to do this in the side-on view due to the curvature between the midbrain and the forebrain.

6. To measure the angle of neural fold elevation, use the *angle tool* in Fiji/ImageJ (Figure 9C). Begin by placing a point at the lateral-most edge of the midbrain–forebrain boundary (the anterior-most position visible in the dorsal view). Place the middle point at the center of the anterior-most midline and the final point at the edge of the other lateral neural fold.

7. The final sample size should be n > 3 embryos (and ideally n > 5 if possible). This is sufficiently powered to account for moderate effect sizes on the order of dozens of microns difference between the mean values of control and experimental conditions. Increased n-values may be required for lower effect sizes.

**Critical:** Close stage-matching is important here, as these values change rapidly during the process of cranial neural tube closure.

8. You can use similar approaches to quantify other aspects of the tissue (e.g., you can find the total area by using the *polygon tool*).

9. To statistically compare width, length, or angle between a control and experimental condition, a simple two-tailed Student’s t-test is usually appropriate. If you have multiple conditions, a one-way ANOVA will likely be a more appropriate test.

**Caution:** In our experience, these morphometric values are normally distributed; when experimental manipulations may alter the distribution of the data, alternative statistical tests would be called for. High variance between individual values in the control group could indicate poor stage-matching between embryos.

**B. Systematic quantitative analysis of apical cell area**

**Figure 10.**
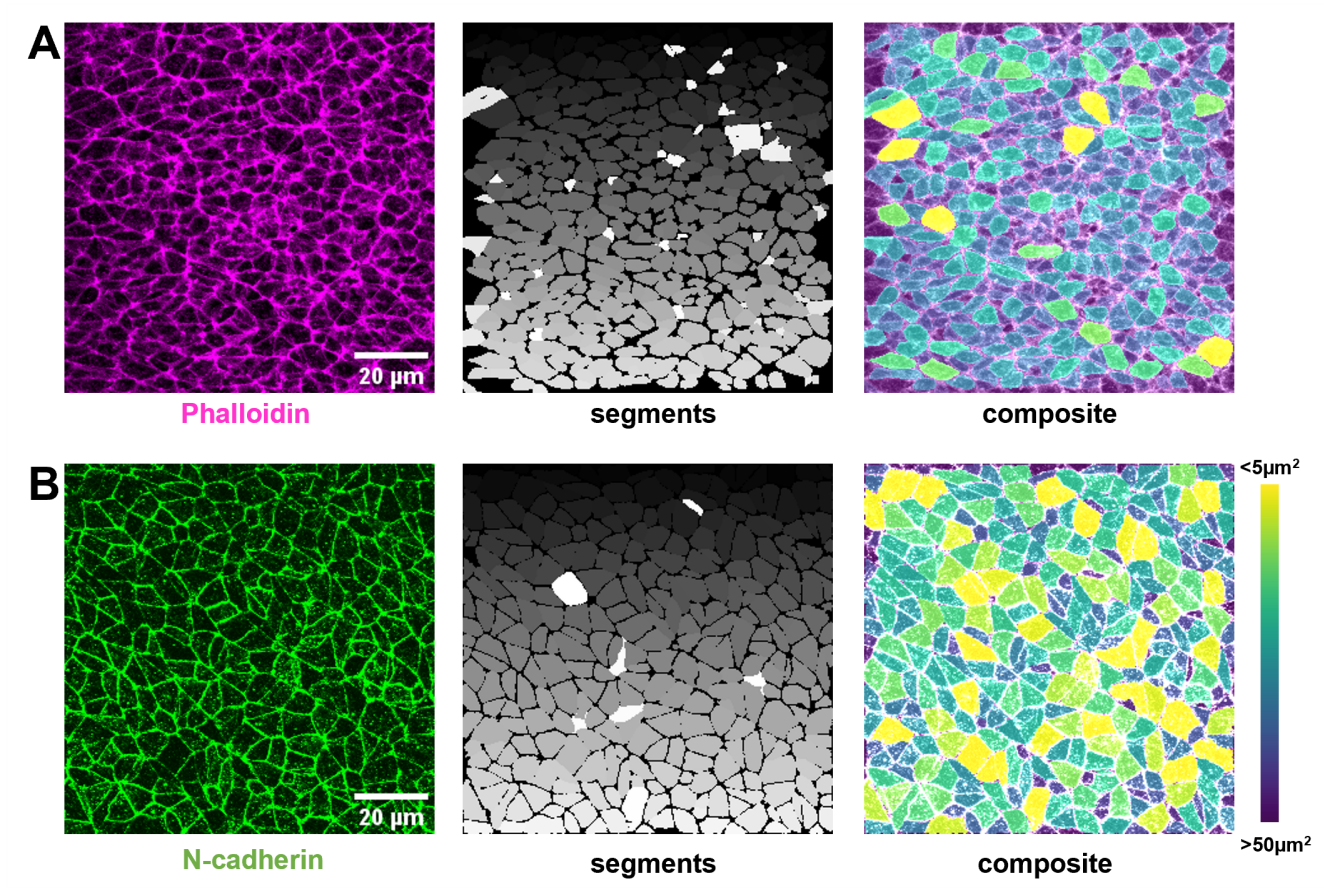
Cell segmentation in the lateral midbrain regions of an 8-somite embryo stained with phalloidin (A) and a 4-somite embryo stained with N-cadherin (B). The raw data, grayscale segments, and composite final segmentation are shown.

*Note: In this section, we will first use segmentation software to define the apical domains of individual cells. We can do this on either the N-cadherin or phalloidin stains after we perform a maximum intensity projection, as both stains are highly enriched specifically at apical junctions and depleted in the cytoplasm (Figure 10). In general, we find that N-cadherin makes for a cleaner segmentation, as the F-actin shows enrichment in the medial domains of cells, as well as heterogeneous cable-like staining along junctions. It is beyond the purview of the current protocol to cover all the various segmentation software that could be used, but we recommend either Cellpose (* *https://www.cellpose.org/* *), which allows the user to train a custom segmentation model and then perform segmentation and manual correction, or SeedWater Segmenter (* *https://github.com/davidmashburn/SeedWaterSegmenter* *), which uses a watershed segmentation algorithm and has an intuitive user interface for correcting the resulting segmentation. Both are freely available and have extensive user documentation to assist in the installation and use of the software. In either case, the reader is encouraged to ensure that their final segmentation is as accurate as possible by closely examining how well individually segmented cells align with the raw data. In Cellpose, a good model to start with for segmenting the apical area in either an N-cadherin or phalloidin stain, is the CP model, which can then be reiteratively trained on your manually corrected segmentations to increase future accuracy of the initial automatic segmentation. The most common automatic segmentation errors are incorrectly merged or split cells, cells with very small apical areas, and dividing cells, and these should be corrected using the user-guided correction tools built into these software packages.*

**Figure 11.**
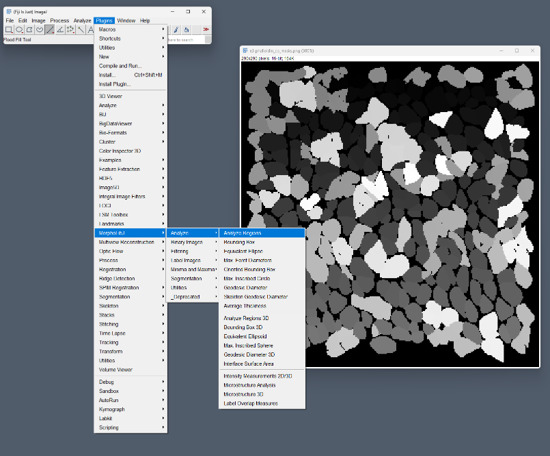
Screenshot of an active segmentation in FIJI/ImageJ demonstrating the menu path to the MorphoLibJ plugin menu. The *Analyze Regions* command is selected.

1. After segmentation is complete, the next steps will be performed in FIJI/ImageJ. First, ensure that the MorphoLibJ plugin (https://github.com/ijpb/MorphoLibJ) is installed. To do so, open FIJI/ImageJ and select the *Update* function under the *Help* menu. Next, click on *Manage Update Sites*. Scroll down the list or use the search bar to find the update site *IJPB-Plugins* and make sure it is active. If it is not already installed, FIJI/ImageJ will prompt you to install it. Be sure to restart the software after the plugin is installed.

2. Open the output image of your segmenter, which consists of a 16- or 32-bit image where each cell segment shares a single gray value (Figure 10). By default, this should be a .png file with a terminal identifier, such as “-masks” at the end of the file name.

3. Because the edges of the cropped region will include incomplete cells, segmentations at the image border should be removed so as not to confound the final analysis. To do so, under the *Plugins* menu, select *MorphoLibJ* and *Label Images*, and then *Remove border labels*.

4. Next, in the ‘MorphoLibJ’ menu, select *Analyze* and then *Analyze Regions* (Figure 11). Make sure the *Area* checkbox is selected. Note that there is a wide variety of additional morphological properties that can be additionally quantified for each segment. Click on any others that are relevant and run the analysis. This will generate a new *Results* window containing the measurements; these can be exported as a CSV spreadsheet for further analysis.

5. (Optional) If you would like to create an image where the apical areas of the cells are color-coded in a heatmap, you can do so by using the *MorphoLibJ* menu and selecting *Assign Measure to Label* under the *Label Images* submenu. Select *Area* as the measure to assign. This can then be recolored in a wide variety of colors using the *Image > Lookup Tables* menu.

6. Once you have collected all the apical areas for each embryo, you can analyze them in two ways. First, you can generate an average apical area value for each embryo. You can then use a simple two-tailed Student’s t-test to compare control embryos (e.g., wild-type) against those of an experimental condition (e.g., mutants). For sufficient power, you will likely need 3–5 embryos, and possibly additional embryos if the effect size is small (<5 μm^2^ difference in means between control and experimental conditions). The second analysis method is to determine the frequency distribution of apical areas by binning areas in 5 or 10 μm^2^ increments and counting the proportion of cells in that bin for each embryo. Control and experimental conditions can then be compared by running a two-way ANOVA test with Sidak’s correction for multiple comparisons, which will reveal any bins that are statistically significant.

**Caution:** In our experience, these morphometric values are normally distributed and exhibit low embryo-to-embryo variance. In cases where experimental manipulations may alter these aspects of the data, alternative statistical tests would be called for.

*Note: For all analyses, the best practice is to compare control and experimental embryos that have been stage-matched to within two somites (e.g., 5–7 somite stages or 7–9 somite stages, not 5–9 somite stages; see notes at the beginning of the Procedure section for information on staging embryos by somite number).*

**Caution:** Two potential sources of high embryo-to-embryo variance within control populations are large stage differences (>2 somites) and a large proportion of segmentation errors. Mutants we have analyzed to date do not show higher variance than controls, but such outcomes would require alternative statistical approaches.

## Validation of protocol

This protocol has been used and validated in the following open-access research articles:

- Brooks et al. [12]. Sonic hedgehog signaling directs patterned cell remodeling during cranial neural tube closure. *eLife* (Figures 1, 2, 4, 5, 8, and 9)
- Bogart and Brooks [13]. Canonical Wnt pathway modulation is required to correctly execute multiple independent cellular dynamic programs during cranial neural tube closure. *Developmental Biology* (Figures 3, 4, 7, and 9)
- Brooks et al. [14]. GPR161-GLI3 repressor signaling at cilia directs apical constriction and cell fate during cranial neural tube closure. *Development* (Figures 7 and 8)

## General notes and troubleshooting

**Troubleshooting**

**Problem 1:** Unstable position of the embryo during brightfield imaging.

Possible cause: Limited support of the embryo by the agarose walls.

Solution: Create divots in the 2% agarose pad approximately the length of an embryo. Then, wedge the embryo between two walls to stabilize it, which allows for greater control of the depth of the whole embryo. This also helps to restrain curled embryos in a flat position.

**Problem 2:** Shifting of the embryo while compressing the cover glass for whole-mount microscopy.

Possible cause: The embryo is sensitive to medium movement and requires stabilization.

Solution: Brace the tips of a pair of forceps at the tail of the embryo, while using the second pair of forceps to carefully tap the cover glass directly above the embryo. Once the embryo is slightly compressed between the coverslip and the cover glass, continue with the outlined protocol above. It is helpful to shake the dish to ensure the embryo is secure and will not dislodge.

**Problem 3:** Over-compression of the embryo.

Solution: To prevent over-compression, we recommend that you only slowly apply increasing pressure during mounting and pause if the embryo begins to flatten. You can test if the mounting pressure is sufficient by gently shaking as above. If the embryo is only mildly over-compressed, you can remove it from under the mounting cover glass and let it rest for 30 min before attempting to remount it.

**Problem 4:** High background noise of fluorescent stains.

Possible cause: Attached amniotic sac in the imaging field.

Solution: Ensure the amniotic sac in the imaging field is removed prior to mounting, as the tissue readily collects secondary antibodies. The amniotic sac of fixed embryos is generally tightly adhered to the embryo; removal from critical areas prior to fixation is recommended.

**Problem 5:** Weak or uneven antibody staining.

Solution: In the case of uneven or weak antibody staining affecting only a single channel, embryos could be restained with just that antibody to attempt to increase the signal. If you continuously observe uneven antibody staining, the embryos could be overfixed, and trying a shorter fixation period or lower temperature may help. If poor fluorescence is observed in multiple channels, consider troubleshooting the staining by, e.g., changing the blocking buffer or antibody concentrations.

**Limitations of this protocol:** The confocal imaging and segmentation portions of this protocol become more difficult or impossible once embryos reach 9–10 somites of age (~E8.75), as the increasing elevation of the neural folds prevents efficient mounting without excessive flattening/compression of the embryo. The brightfield morphometric approaches will still work at these later stages. Additionally, as written, this protocol does not work for subcellular level segmentation. However, the staining and imaging parts can be adapted for such approaches with appropriate changes to the computational analysis pipeline (e.g., [13,20]).
